# Perioperative Outcomes of Robotic Radical Prostatectomy with Hugo™ RAS versus daVinci Surgical Platform: Propensity Score-Matched Comparative Analysis

**DOI:** 10.3390/jcm13113157

**Published:** 2024-05-28

**Authors:** Carlo Gandi, Filippo Marino, Angelo Totaro, Eros Scarciglia, Fabrizio Bellavia, Riccardo Bientinesi, Filippo Gavi, Pierluigi Russo, Mauro Ragonese, Giuseppe Palermo, Marco Racioppi, Nicolò Lentini, Roberta Pastorino, Emilio Sacco

**Affiliations:** 1 Department of Urology, Fondazione Policlinico Universitario Agostino Gemelli IRCCS, 00168 Rome, Italy; carlo.gandi@guest.policlinicogemelli.it (C.G.); angelo.totaro@policlinicogemelli.it (A.T.); eros.scarciglia@guest.policlinicogemelli.it (E.S.); fabrizio.bellavia01@icatt.it (F.B.); riccardo.bientinesi@policlinicogemelli.it (R.B.); filippo.gavi@guest.policlinicogemelli.it (F.G.); pierluigi.russo1@guest.policlinicogemelli.it (P.R.); mauro.ragonese@policlinicogemelli.it (M.R.); giuseppe.palermo@policlinicogemelli.it (G.P.); marco.racioppi@policlinicogemelli.it (M.R.); 2 Department of Medicine and Translational Surgery, Università Cattolica Del Sacro Cuore, 00168 Rome, Italy; emilio.sacco@unicatt.it; 3 Department of Urology, Addenbrooke’s Hospital, Cambridge University Hospitals NHS Foundation Trust, Cambridge CB2 0QQ, UK; 4 Department of Life Sciences and Public Health, Section of Hygiene, Università Cattolica del Sacro Cuore, 00168 Rome, Italy; nicol.lentin@libero.it (N.L.); roberta.pastorino@unicatt.it (R.P.); 5 Department of Woman and Child Health and Public Health—Public Health Area, Fondazione Policlinico Universitario Agostino Gemelli IRCCS, 00168 Rome, Italy; 6 Department of Urology, Ospedale Isola Tiberina—Gemelli Isola, 00168 Rome, Italy

**Keywords:** robot-assisted radical prostatectomy, Hugo^TM^ RAS system, daVinci surgical platform, robotic surgery, comparative outcomes, prostate cancer

## Abstract

**Background/Objectives**: There is an urgent need for comparative analyses of the intraoperative, oncological, and functional outcomes of different surgical robotic platforms. We aimed to compare the outcomes of RARP performed at a tertiary referral robotic centre with the novel Hugo^TM^ RAS system with those performed with a daVinci surgical system, which is considered the reference standard. **Methods**: We analysed the data of 400 patients undergoing RARP ± pelvic lymph node dissection between 2021 and 2023, using propensity score (PS) matching to correct for treatment selection bias. All procedures were performed by three surgeons with Hugo^TM^ RAS or daVinci. **Results**: The PS-matched cohort included 198 patients with 99 matched pairs, balanced for all covariates. Positive surgical margins (PSMs) were found in 22.2% and 25.3% (*p* = 0.616) of patients, respectively, in the Hugo^TM^ RAS and daVinci groups. No significant differences were found for other important perioperative outcomes, including median (1st–3rd q) operative time (170 (147.5–195.5) vs. 166 (154–202.5) min; *p* = 0.540), median (1st–3rd q) estimated blood loss (EBL) (100 (100–150) vs. 100 (100–150) ml; *p* = 0.834), Clavien–Dindo (CD) ≥ 2 complications (3% vs. 4%; *p* = 0.498), and social continence at 3 months (73.7% vs. 74.7%; *p* = 0.353). In multiple analyses, no associations were found between surgical outcomes (PSM, length of PSM, operative time, EBL, length of catheterization, length of hospital stay, social continence at three months after surgery, and CD ≥ 2 complications) and the robotic platform. **Conclusions**: Our findings demonstrate that Hugo^TM^ RAS enables surgeons to safely and effectively transfer the level of proficiency they reached during their previous experience with the daVinci systems.

## 1. Introduction

Robot-assisted radical prostatectomy (RARP) is the most adopted treatment for localized prostate cancer [1]. After receiving Food and Drug Administration (FDA) approval for prostate surgery in May 2001, the daVinci surgical system (Intuitive Surgical Inc., Sunnyvale, CA, USA) contributed to the widespread diffusion of robotic prostate surgery worldwide, with proven benefits for the surgeon (including improved three-dimensional visualization, magnification, and articulated wristed instruments with tremor filtering and motion scaling) and the patient (reduced postoperative pain, less blood loss, and shorter hospital stay) [2]. As of December 2023, Intuitive Surgical sold 8606 daVinci robotic surgical systems worldwide [3].

Despite these benefits, the undeniably higher costs have hindered the diffusion of robotic surgery as a standard of care compared to open and, especially, laparoscopic surgery [4]. The cost issue was also linked to the lack of competitors due to the patents owned by Intuitive, some of which have expired since 2019, allowing other manufacturers to introduce novel robotic surgical platforms [5]. As a result, we are facing a major transformation in the robotic surgery landscape, which currently includes several systems, of which two obtained the CE (Conformité Européenne) mark approval: Versius™ (CMR, Cambridge, UK) and Hugo™ RAS (Medtronic, Minneapolis, MN, USA).

The Hugo™ RAS received CE mark approval for urological procedures in adults in October 2021. It consists of a multi-port modular robotic platform bearing innovative features such as an open console with a novel hand controller design, and independent arm-carts [6].

Several authors showed the feasibility of RARP performed with Hugo™ RAS and with promising perioperative outcomes [6,7,8,9,10,11,12]. Nevertheless, due to its wide and long-standing use and the huge body of published evidence, RARP performed with the daVinci surgical system (daVinci–RARP) remains the reference standard. Therefore, to fully understand the potential of the Hugo™ RAS system in performing RARP (Hugo–RARP), more reliable literature comparing the outcomes of Hugo–RARP and daVinci–RARP is required. Therefore, in this study, we aimed to compare the perioperative outcomes of Hugo–RARP vs. daVinci–RARP at a high-volume tertiary referral centre, the first in Italy using Hugo™ RAS in urological surgery, by a propensity score matching (PS-matching) analysis [13].

## 2. Material and Methods

### 2.1. Patient Population and Study Design

All consecutive patients undergoing RARP at Fondazione Policlinico Universitario A. Gemelli IRCCS (Rome, Italy) between September 2021 and September 2023 were screened for inclusion in this IRB-approved observational comparative study (ID 5119/2022). Prospectively collected patients’ data were retrospectively analysed. The inclusion criteria were (1) RARP is performed with the Hugo™ RAS system or daVinci Xi system; (2) the operator is one of three experienced surgeons who already achieved stable surgical margin proficiency with the daVinci Xi system [14], and who has a caseload of over 500 RARPs performed with the daVinci Xi system; (3) informed consent is signed. Exclusion criteria were missing data (e.g., lack of magnetic resonance imaging data), conversion to open surgery, and the assumption of preoperative hormonal therapy. After March 2022, both Hugo™ RAS and daVinci robotic systems were used at our institution without any specific preference to adopt one platform over the other. However, a learning curve was expected for Hugo–RARP; thus, patients who had a high-risk disease, which required pelvic lymph node dissection; who had previous major abdominal surgery; and who received a trans-urethral resection of the prostate (TURP) or neoadjuvant hormone therapy were excluded from the initial series of ten cases. Consequently, patient inclusion for the Hugo^TM^ arm commenced from the eleventh case performed at our hospital, without applying any selection criteria.

The research adhered to the Strengthening the Reporting of Observational Studies in Epidemiology (STROBE) guidelines for reporting observational studies (Supplementary Materials).

### 2.2. Technical Insights of Hugo™ RAS and daVinci Systems

The new Hugo^TM^ RAS system consists of one system tower with a Valleylab^®^ electrosurgical generator, four independent arm carts, and an open console. In contrast to the daVinci system’s closed console, which isolates surgeons from the environment during procedures, aiming to reduce susceptibility to external distractions, Hugo^TM^’s open console intends to promote heightened awareness of the operating room. This design aims to foster greater multitasking and direct communication within the surgical team. Additionally, Hugo^TM^’s ergonomic console design allows for improved surgeon positioning, offering greater freedom of head movement and the ability to adopt various postures during surgery [15,16].

Both surgical platforms offer 3D HD vision but based on different technologies. While the daVinci system uses stereoscopic technology with two monitors, one for each eye of the operator, transmitting the images of an Intuitive^TM^ proprietary 3D endoscope, Hugo^TM^ features a passive 3D display requiring specific tracker-equipped glasses and reproducing the images coming from a Karl Storz 3D laparoscopic endoscope [17,18,19].

Regarding instrument control, the Hugo^TM^ console has “pistol-like” hand controllers that offer a scaling factor for wrist rotation up to 2x and a rotation range up to 520°, facilitating operation such as suturing in deep cavities. Both systems offer tremor filtering to improve movement precision, but with differences in pedal controls. While the daVinci system allows for instrument change with a single click of the pedal, Hugo^TM^ requires the pedal to be held down for 1.5 s to effect the change [15,16,17,18].

Additionally, the Hugo^TM^ platform lacks some advanced instruments available in the daVinci system, such as rapid sealing devices and clip appliers [15,16].

Both platforms feature an artificial intelligence (AI) tool: MyIntuitive software for the daVinci system and Touch Surgery^TM^ software for the Hugo^TM^ platform. However, the Hugo^TM^ software has additional functionality: leveraging AI algorithms, the system automatically segments videos into procedural steps, offering a tool to improve the performance of experienced surgeons and facilitate the training of younger surgeons (streamlining teaching) [17,18].

### 2.3. Surgical Technique

All RARP procedures were performed according to the well-standardized Montsouris technique [14,20] for laparoscopic prostatectomy adapted to the robotic approach. Our previous work describes the technique in detail as per port placement and docking settings [6]. The instruments routinely used with the Hugo^TM^ RAS system were monopolar curved scissors, bipolar Maryland forceps, Cadière forceps, and large needle drivers [6]. Otherwise, Prograsp forceps were used instead of Cadière with the daVinci platform.

### 2.4. Measurements and Outcomes

Patients’ demographic and clinical data were obtained from the prospectively maintained database. We assessed the positive surgical margin (PSM) rate as a primary outcome, defined as tumour cells abutting the inked surgical margins of the specimen [21]. Whole-mount sections were prepared at 5 mm sections and stained with haematoxylin and eosin for histological evaluation. Immunohistochemical studies were performed to improve the assessment of crush artefacts derived from the electrocauterization [22].

PSM locations have been classified into apex, right (RPL), and left (LPL) posterolateral, bladder neck (BN), according to a multi-institutional study by Patel et al. [23]. Multifocal PSMs or PSMs longer than 3 mm (MF/> 3 mm) were also recorded [24]. The International Society of Urological Pathology (ISUP) grade on biopsy was classified into two categories: 1–3 versus 4–5. The Charlson Comorbidity Index (CCI) was categorized as CCI 1–2, CCI 3–4, and CCI ≥ 5.

Secondary outcomes included the operative time, intra- and postoperative complications, estimated blood loss (EBL), length of catheterization, length of hospital stay (LOS), number of lymph nodes removed, and postoperative continence status. Follow-up visits were performed at 3 and 6 months from surgery. A successful functional outcome was defined as achieving a cure (no pad use) or social continence (use of no more than one pad per day) [25].

Furthermore, through a semistructured questionnaire (Figure S1), we evaluated users’ satisfaction with each Hugo^TM^ robotic instrument when compared to their experience with daVinci’s instruments.

## 3. Statistical Methods

Descriptive statistics were employed, with continuous variables summarized using the median and the first and third quartile (q1–q3) values and categorical variables expressed as absolute and percentage frequencies. Continuous variables were compared using either the independent *t*-test or Mann–Whitney U-test, depending on data distribution [26]. Categorical variables were compared using the Chi-squared or Fisher’s exact test, as appropriate.

A PS-matching analysis addressed potential biases in preoperative patient characteristics. PS-matching represents an alternative approach to treatment–effect estimation by considering the conditional probability of treatment selection [27]. As a balancing score, the propensity score is the probability of treatment assignment for an individual, contingent on observed covariates. The distribution of measured baseline covariates is comparable between treated and untreated subjects, contingent on the propensity score; this facilitates the mitigation of bias when comparing interventions across treatment groups [27]. Propensity scores were computed using a logistic regression model based on covariates such as age, BMI, prostate volume, preoperative PSA level, biopsy ISUP grade, previous abdominal surgery, and CCI. PS-matching was conducted in a 1:1 ratio using a greedy nearest-neighbour algorithm, with a calliper width set at 0.2 standard deviations of the logit of the propensity score and without replacement. Covariate balance was evaluated by comparing the baseline covariates and of the cumulative distribution functions of the propensity scores of each matched sample [28].

Both *p*-values and standardized mean difference (SMD) were used to compare variables between treatment groups [28]. To achieve a good matching balance, the absolute value of SMD was preferentially <0.1 [29].

Additionally, we conducted a sensitivity analysis of the ignorability assumption under PS-matching. This assumption implies that all variables that impact the treatment assignment and outcome have been observed and measured. If unobserved factors influence both treatment assignment and the outcome variables, our estimated effects may be biased (hidden bias). We used Rosenbaum’s bounding approach [13,30,31] in order to test whether our results were sensitive to such unobserved heterogeneity. This approach involves one sensitivity parameter (Γ ≥ 1) that indicates the association (odds) of an unobserved variable with treatment assignment (the higher the value of Γ, the lower the sensitivity of the study to unmeasured confounders).

Logistic and linear regression models were constructed to explore the association between the robotic system and surgical outcomes in the matched population.

Furthermore, a sensitivity analysis was conducted to examine the association between the robotic system and surgical outcomes in the unmatched population. This analysis employed adjusted multiple regression models. Adjustments for casemix included variables used in the propensity score, such as age, BMI, prostate volume, preoperative PSA level, biopsy ISUP grade, previous abdominal surgery, and CCI.

Effect sizes were reported as odds ratios (ORs) or Beta coefficients along with their 95% confidence intervals (CIs). A two-sided *p*-value < 0.05 was considered statistically significant.

All statistical analyses were executed using R version 4.2.0 (2022-04-22) for Windows, and the MatchIt package from the R Foundation for Statistical Computing facilitated the PS-matching analysis. We sticked to the published guidelines on reporting studies with propensity score matching (Supplementary Table S1) [32].

## 4. Results

### 4.1. Matching Procedure and Baseline Characteristics

**Figure 1:**
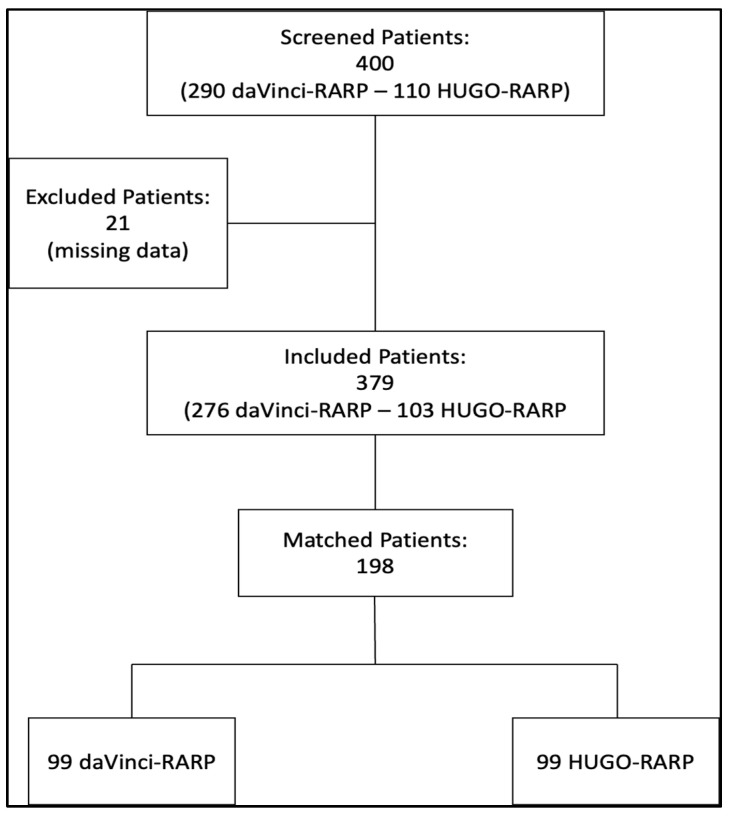
Study flowchart.

Out of 400 screened patients, 379 remained eligible after patients with missing data were removed—276 in the daVinci–RARP group and 103 in the Hugo–RARP group. Subsequently, 198 (52.2%) were matched according to the propensity score (Figure 1).

Table 1 describes the baseline characteristics of the patients in the unmatched and matched population, grouped by the surgical platform used.

Patients in the Hugo–RARP group showed a lower rate of preoperative CCI ≤ 2 (2.9% vs. 12.3%; *p* < 0.001) and a higher rate of CCI ≥ 5 (57.3% vs. 30.1%; *p* < 0.001) before PS-matching. No statistically significant differences were observed between the two groups for all matched variables based on *p*-values.

**Figure 2:**
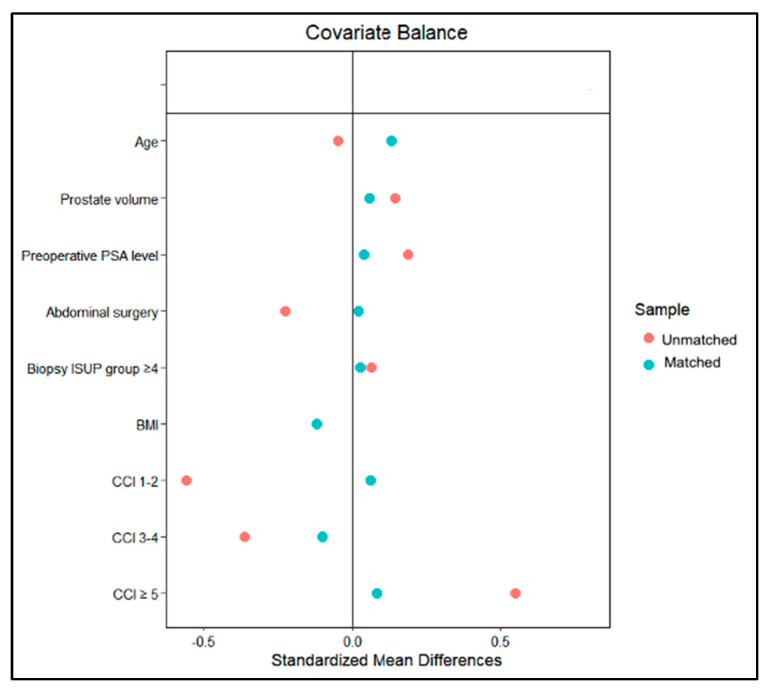
Covariate balance between Hugo–RARP and daVinci–RARP patients.

However, upon diagnostic graph assessment of covariate balance (Figure 2), a satisfactory balance in PS-matching of covariates was shown between Hugo–RARP and daVinci–RARP patients, except for age and BMI, which were slightly above the 0.1 cutoff point for the SMD (0.136 and −0.122, respectively). Nonetheless, these differences were considered nonsignificant, taking into account the *p*-values.

**Figure 3:**
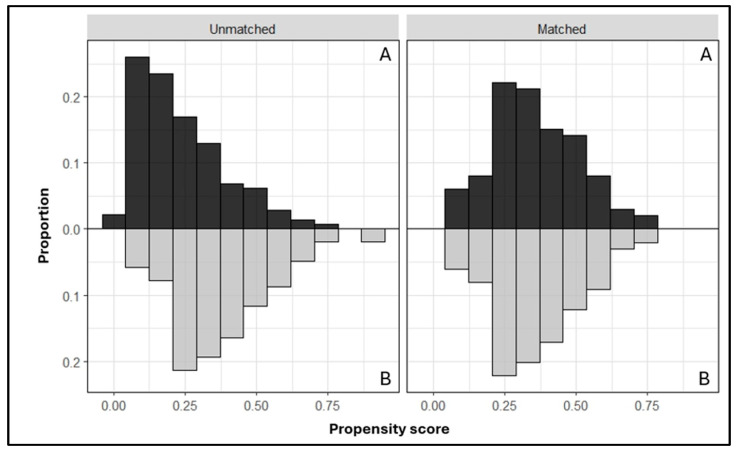
Assessing common support assumption required for propensity score matching procedure (A = daVinci–RARP group; B = Hugo–RARP).

Accordingly, a satisfactory degree of overlap in the propensity score between groups was observed (Figure 3), and the SMD in the propensity score between matched subjects was 0.023.

### 4.2. Primary Outcome

Table 2 summarizes intra- and postoperative outcomes in matched and unmatched populations grouped by the surgical platform used.

In the daVinci–RARP and Hugo–RARP groups, 25 (25.3%) and 22 (22.2%) patients had PSMs on final pathology, respectively, with no significant differences between the two platforms (*p* = 0.616).

#### Focus on Positive Surgical Margins

No statistically significant differences were found in terms of PSM locations (all *p* > 0.1). Table 3 summarizes the distribution of PSMs in the matched population. Furthermore, in the model analyses (Table 4) among the propensity score-matched cohort, no statistically significant differences were observed regarding PSMs (OR: 0.846, 95% CI: 0.439–1.629; *p* = 0.616).

### 4.3. Secondary Outcomes

As shown in Table 2 and Table 4, within the PS-matched cohort, no statistically significant differences were observed between daVinci and Hugo™ patients in terms of bilateral nerve-sparing and pelvic lymphadenectomy rates. However, it is noteworthy that, even if there was no statistical significance, the rate of patients undergoing lymphadenectomy was evidently higher in the daVinci group (33.6% vs. 23.7%, *p* = 0.06), although the mean number of nodes removed does not show macroscopic differences between the two surgical platforms (3.56 vs. 2.38, *p* = 0.09). Final pathology also revealed no significant differences in the pT stage and node stage. Additionally, there were no significant differences between the two groups in terms of the length of hospitalization stay, length of catheterization, operative time, EBL, Clavien–Dindo ≥ 2 complication rate, and social continence status at three months after surgery.

### 4.4. Users’ Satisfaction with Hugo^TM^’s and daVinci’s Instruments

In regards to monopolar scissors, two out of three surgeons reported a similar experience (3/5) between Hugo^TM^ RAS and daVinci, while one surgeon reported a better experience (4/5) with the Hugo^TM^ RAS scissors, which offers a more effective cold cut of tissue compared to the daVinci scissors.

No differences were reported for the Maryland bipolar forceps and the Hugo^TM^ RAS needle driver, as all three surgeons found them to provide a similar experience (3/5) compared to the instruments of the daVinci system.

The greatest differences emerged for the Hugo^TM^ RAS Cadière forceps, which were found by all three surgeons to provide a worse experience (2/5) than daVinci due to its limited grasping capacity.

### 4.5. Sensitivity Analysis

Table 5 shows the results of the sensitivity analysis for the primary outcome. It checks that the impact of the potential unmeasured confounding agent on the probability of assignment between Hugo^TM^ RAS and daVinci surgical systems does not significantly influence the estimates derived from the analysis (i.e., does not change the inference found). The platform effect turns insignificant at a critical Γ value of 1.55. This means that our study is insensitive to unmeasured potential confounders that increase the probability of being operated on with Hugo^TM^ RAS by up to 55%. We can conclude that this study is reasonably robust to unobserved heterogeneity.

Table 6 summarizes the results of the sensitivity analysis for perioperative outcomes in the unmatched population. No significant differences in outcome results were observed compared to those identified in the matched analysis.

## 5. Discussion

To the best of our knowledge, this is the first analysis comparing early perioperative outcomes of patients undergoing RARP with Hugo™ RAS and daVinci surgical robotic systems using a quasi-randomized study design based on propensity score matching (Canadian task force classification II-2).

After adjusting for potential treatment selection bias, no significant differences were found in terms of the PSM rate between the two surgical platforms. Interestingly, in our study, the PSM rates of the two platforms were comparable at the level of all the margin locations. Of note, multifocal PSM rates that are associated with a recognized impact on the risk of biochemical recurrence (BCR) [33] did not differ significantly between Hugo^TM^ RAS and daVinci procedures. These findings together suggest that oncological safety was maintained during the introduction of Hugo™ RAS into clinical practice.

All surgeons who performed the procedures reported some degree of dissatisfaction with the lower traction ability of the Hugo™ RAS Cadière forceps compared to the daVinci Prograsp forceps. However, this difference did not appear to affect the PSM rate nor in the posterolateral prostatic location as well. By the way, a new Hugo™ RAS Cadière forcep with increased gripping force has been anticipated (Secure Cadière) by Medtronic. Curiously, although in the absence of statistical significance, the PSM rate in the posterolateral location was even higher in the daVinci population, with a more marked difference observed in the left posterolateral location. Similarly, the rate of multifocal PSMs/>3 mm was also evidently higher for the daVinci group, albeit again without statistical significance. However, these differences could be the result of the limited sample size and require further study in a larger series.

In patients receiving RARP, the two robotic platforms also performed similarly for all other perioperative outcomes. In particular, no statistically significant differences emerged in terms of surgical complications, EBL, operative time, LOS, and length of catheterization. As argued by Bravi et al., it is reasonable to assume that daVinci procedures might be considered the reference for several RARP surgical outcomes, including operative time [12]. It is noteworthy that, despite Hugo^TM^’s apparently more complex and time-consuming docking system due to its independent arm-cart configuration, in our study, Hugo^TM^ RAS operative time did not differ significantly from daVinci procedures. Comparable outcomes between the two platforms were also found for continence recovery at three months.

Furthermore, through multiple models, we could also assess that there was no association between the use of a specific robotic platform and important perioperative outcomes such as PSMs, EBL, and operative time, reinforcing the results emerging from the propensity score matching analysis. These findings were consistent with the large RARP series published by Bravi et al. [12] as well as with the smaller initial series of Ragavan et al. [9] and Hsien-Che Ou et al. [19], who curiously reported, for Hugo^TM^ RAS, a more time-consuming vesicourethral anastomosis, which they attributed to a trocar malpositioning. In addition to the aforementioned publications, our comparative analysis of a large multisurgeon series and the reliability of its results were strengthened by the use of the PS-matching methodology, making the groups under comparison uniform in relation to major potential treatment-selection biases in a quasi-randomized fashion.

It is not to be overlooked that all surgeons involved in our study received a dedicated dry and wet lab training on the Hugo™ RAS docking system and console controls at ORSI Academy (Melle, Belgium). Team-based training, also involving bed side assistants and scrub nurses, was conducted in order to standardize port placement and arms’ configuration sequence, also improving troubleshooting skills. Furthermore, almost all procedures took place in the presence of a Medtronic technician in the operating room, supervising the correct functioning of the robotic system within our centre. These conditions certainly helped the transition to this new platform.

In agreement with our findings but outside the context of RARP, in a comparative analysis of robot-assisted simple prostatectomy (RASP), Balestrazzi et al. [34] found no significant differences between Hugo^TM^ RAS and daVinci procedures in terms of perioperative outcomes, as is the case for robot-assisted sacrocolpopexy (RASC) in the work of Collà Ruvolo et al. [35].

The strengths of our study stay in the large multisurgeon series analysed with the use of the PS-matching methodology, making the groups under comparison uniform in relation to major potential selection biases and ensuring high-quality reporting of observational studies. However, the prediction probabilities of the logistic regression model may have been biased due to the data imbalance in the ratio of Hugo–RARP to daVinci–RARP, which was 1:1 in the matched population and 1:2.6 in the unmatched population. Limitations of this study also include its retrospective nature, even if based on a prospectively collected database. Furthermore, erectile function and postoperative PSA values were not included among the evaluated outcomes, although we plan to update our results with more meaningful data on functional and oncological outcomes when a longer-term follow-up will be reached in our population. We analysed the PSM rate as a commonly used surrogate endpoint for tumour recurrence in that a two- to five-fold higher risk of biochemical recurrence is reported in men with PSMs compared to those with negative margins [36].

Based on all the acquired evidence, we can say that Hugo™ RAS allows surgeons, with prior robotic experience on the daVinci surgical system, to safely transfer their surgical skills to a new robotic platform, without jeopardizing their proficiency status. However, subsequent studies with extended follow-up are needed to evaluate if the use of a different robotic surgical system might affect long-term oncological and functional outcomes. Additionally, conducting a cost comparison between surgeries carried out using the daVinci and Hugo™ RAS surgical systems can offer valuable perspectives for healthcare decision-makers.

## Figures and Tables

**Table 1:** Comparison of baseline characteristics between groups in the unmatched and matched populations.

	Unmatched Population				Matched Population			
Median (1st–3rd q) or n (%)	daVinci (N = 276)	Hugo (N = 103)	SMD	*p*-Value	daVinci (N = 99)	Hugo (N = 99)	SMD	*p*-Value
Age (years)	68 (63–72)	68 (62–72)	−0.0495	0.758	67 (62–71)	68 (62.5–72)	0.1355	0.259
Prostate volume (mL)	45.00 (31.60–59.25)	48.00 (34.00–64.00)	0.1473	0.147	45.00 (32.00–61.00)	50.00 (34.00–64.50)	0.0571	0.473
Preoperative PSA level (ng/mL)	7.50 (5.60–10.03)	7.89 (5.50–11.82)	0.1918	0.272	8.00 (5.90–11.65)	7.70 (5.44–10.05)	0.0380	0.735
BMI (kg/m^2^)	26.46 (24.69–28.65)	26.00 (24.25–28.00)	−0.1210	0.218	27.04 (25.00–28.38)	26.00 (24.25–28.00)	−0.1219	0.304
Biopsy ISUP group ≥ 4	39 (14.1%)	17 (16.5%)	0.0640	0.626	15 (15.2%)	16 (16.2%)	0.0272	1
Abdominal surgery	132 (47.8%)	38 (36.9%)	−0.2266	0.064	37 (37.4%)	38 (38.4%)	0.0209	1
Charlson Comorbidity Index				<0.001				0.724
1–2	34 (12.3%)	3 (2.9%)	−0.5594		2 (2.0%)	3 (3.0%)	0.0601	
3–4	159 (57.6%)	41 (39.8%)	−0.3637		45 (45.5%)	40 (40.4%)	−0.1032	
≥5	83 (30.1%)	59 (57.3%)	0.5500		52 (52.5%)	56 (56.6%)	0.0817	

SMD = standardized mean difference; BMI = body mass index; ISUP = International Society of Urological Pathology; PSA = prostate-specific antigen.

**Table 2:** Comparison of intra- and postoperative outcomes between groups in the unmatched and matched population.

	Unmatched Population				Matched Population			
Median (1st–3rd q) or n (%)	daVinci (N = 276)	Hugo (N = 103)	SMD	*p*-Value	daVinci (N = 99)	Hugo (N = 99)	SMD	*p*-Value
Positive surgical margins	58 (21.0%)	23 (22.3%)	0.0316	0.781	25 (25.3%)	22 (22.2%)	−0.0728	0.616
Length of positive surgical margins (mm) *	12.5 (6.0–19.8)	13.0 (8.0–25.5)	0.2236	0.419	12.0 (6.0–23.0)	12.0 (8.0–23.8)	−0.0197	0.685
Operative time (min)	170.0 (147.0–206.0)	170.0 (147.5–195.5)	0.0379	0.956	166 (145–202.5)	170 (147.5–195.5)	0.1100	0.540
Estimated blood loss (mL)	100 (100–155)	100 (100–150)	0.0294	0.653	100 (100–150)	100 (100–150)	0.0600	0.834
Length of hospital stay (days)	4 (4–5)	4 (4–5)	−0.0535	0.400	4 (4–5)	4 (4–5)	−0.2263	0.268
Length of catheterization (days)	17 (15–20)	15 (14–20.5)	−0.0036	0.407	17 (15–20)	15 (14–20)	−0.0326	0.473
Clavien–Dindo ≥ 2 complications	14 (5.1%)	3 (2.9%)	−0.1284	0.577	6 (4.0%)	3 (3.0%)	−0.1802	0.498
Social continence at 3 months after surgery	202 (73.2%)	78 (75.7%)	−0.0795	0.481	73 (73.7%)	74 (74.7%)	−0.1320	0.353
Nerve-sparing surgery				0.520				0.844
No nerve-sparing	109 (39.5%)	44 (42.7%)	0.0323		39 (39.4%)	42 (42.4%)	0.0303	
Nerve-sparing bilateral	100 (36.2%)	31 (30.1%)	−0.0613		34 (34.3%)	30 (30.3%)	−0.0404	
Nerve-sparing unilateral	67 (24.3%)	28 (27.2%)	0.0291		26 (26.3%)	27 (27.3%)	0.0101	
Pathological stage T at final histology				1				0.625
pT2	2 (0.7%)	0 (%)	−0.1000		0 (0%)	0 (0%)	0	
pT2a	6 (2.2%)	2 (1.9%)	−0.0168		2 (2.0%)	2 (2.0%)	0	
pT2b	2 (0.7%)	0 (0%)	−0.1000		1 (1.0%)	0 (0%)	−0.1394	
pT2c	198 (71.7%)	76 (73.8%)	0.0466		66 (66.6%)	73 (73.7%)	0.1608	
pT3a	34 (12.3%)	13 (11.7%)	0.0091		13 (13.1%)	13 (13.1%)	0	
pT3b	34 (12.3%)	12 (11.7%)	−0.0208		17 (17.2%)	11 (11.1%)	−0.1889	
Pelvic lymphadenectomy	94 (34.0%)	26 (25.3%)	−0.0870	0.053	34 (33.6%)	24 (23.7%)	−0.1000	0.061
Mean (± SD) number of nodes removed ^§^	4.15 (± 6.39)	2.38 (±4.22)	−1.7700	0.010	3.56 (±5.47)	2.38 (± 4.27)	−1.1800	0.092
Pathological stage N at final histology ^§^				0.394				0.143
pN0	85 (90.4%)	23 (88.5%)	−0.0210		28 (82.4%)	22 (91.7%)	0.1128	
pN1	9 (9.6%)	3 (11.5%)	−0.1979		6 (17.6%)	2 (8.3%)	−1.1055	

* In patients with positive surgical margin. ^§^ In patients who underwent pelvic lymphadenectomy.

**Table 3:** Focus on positive surgical margin locations.

	Matched Population		
Number (%)	daVinci (N = 99)	Hugo (N = 99)	*p*-Value
PSM	25 (25.2%)	22 (22.2%)	0.616
Apex	12 (12.12%)	8 (8.08%)	0.345
Bladder neck	7 (7.07%)	6 (6.06%)	0.774
Posterolateral	16 (16.16%)	10 (10.10%)	0.206
Right-PL	6 (6.06%)	4 (4.04%)	0.516
Left-PL	10 (10.01%)	6 (6.06%)	0.296
Multifocal or >3 mm	14 (14.14%)	8 (8.08%)	0.174

PSM = positive surgical margin; PL = posterolateral.

**Table 4:** Model analyses exploring the association between the robotic system used (Hugo vs. daVinci) and intra-postoperative outcomes in propensity score-matched cohort ^a,b^.

N = 198	OR/Beta (95%CI) Hugo (1) vs. daVinci (0)	*p*-Value
Positive surgical margins	0.846 (0.439, 1.629)	0.616
Length of positive surgical margins (mm) *	−0.247 (−8.90, 8.41)	0.954
Operative time (min)	5.030 (−7.08, 17.14)	0.417
Estimated blood loss (mL)	6.566 (−19.48, 32.61)	0.622
Length of catheterization (days)	−0.293 (−2.91, 2.32)	0.825
Length of hospital stay (days)	−0.404 (−1.13, 0.32)	0.276
Social continence at 3 months after surgery	0.718 (0.352, 1.439)	0.354
Clavien–Dindo ≥ 2 complications	0.484 (0.100, 1.892)	0.315

OR = odds ratio; CI = confidence interval. ^a^ Patients who underwent daVinci are the reference group. ^b^ Propensity scores were estimated based on age, body mass index, prostate volume, preoperative PSA level, biopsy ISUP group, previous abdominal surgery, and Charlson Comorbidity Index. * In patients with positive surgical margins.

**Table 5:** Sensitivity analysis for the primary outcome variable.

Gamma Values (Γ)	Rosenbaum’s Lower Bound Two-Tailed *p*-Value
1.00	0.4869
1.05	0.4029
1.10	0.3316
1.15	0.2716
1.20	0.2215
1.25	0.1801
1.30	0.1460
1.35	0.1182
1.40	0.0955
1.45	0.0770
1.50	0.0621
**1.55**	**0.0501**
1.60	0.0403

Γ: odds of differential assignment to HUGO due to an unobserved factor. In a study free of hidden bias, Γ is equal to 1. With increasing Γ, the lower bound decreases. The Γ and lower bound *p*-value for the desired significance level (*p* > 0.05) are in bold.

**Table 6:** Sensitivity analysis utilizing multiple regression models to explore the association between the robotic system used (Hugo vs. daVinci) and intra-postoperative outcomes in unmatched population ^a,b^.

N = 379	OR/Beta (95%CI) Hugo (1) vs. daVinci (0)	*p*-Value
Positive surgical margins	0.851 (0.463, 1.564)	0.603
Length of positive surgical margins (mm) *	2.616 (−5.71, 10.94)	0.541
Operative time (min)	0.039 (−10.22, 10.22)	0.994
Estimated blood loss (mL)	2.939 (−18.79, 24.67)	0.791
Length of catheterization (days)	0.518 (−1.63, 2.67)	0.636
Length of hospital stay (days)	−0.223 (−0.74, 0.29)	0.396
Social continence at 3 months after surgery (N = 355)	0.829 (0.456–1.532)	0.545
Clavien–Dindo ≥ 2 complications	0.388 (0.077, 1.390)	0.185

OR = odds ratio; CI = confidence interval. ^a^ Patients who underwent daVinci are the reference group. ^b^ Models were adjusted for age, body mass index, prostate volume, preoperative PSA level, biopsy ISUP group, previous abdominal surgery, and Charlson Comorbidity Index. * In patients with positive surgical margins.

## Data Availability

The data presented in this study are available on request from the corresponding author.

## Supplementary Materials

The following supporting information can be downloaded at: https://www.mdpi.com/article/10.3390/jcm13113157/s1, Table S1: Full guidelines for reporting propensity score analysis, modified from the STROBE (Strengthening the Reporting of Observational Studies in Epidemiology) Statement. Figure S1: Users’ satisfaction with Hugo^TM^ and daVinci instruments. Ref. [37] is cited in the Supplementary Materials.
