# Supplementary material for: Perioperative Outcomes of Robotic Radical Prostatectomy with Hugo™ RAS versus daVinci Surgical Platform: Propensity Score-Matched Comparative Analysis
Source: J Clin Med. 2024 May 28;13(11):3157. doi: 10.3390/jcm13113157 (PMC11173080; doi:10.3390/jcm13113157)
Supplement: Supplementary file 1 [file jcm-13-03157-s001.zip › jcm-2964710-supplementary.pdf]

Compared to your experience with daVinci's instruments, how would you rate your experience with HUGO's instruments

Monopolar Scissors		
1	much worse	☐
2	worse	☐
3	equal	☐
4	better	☐
5	much better	☐
Please justify your answer by indicating PROs and CONs		

Bipolar Maryland Forceps		
1	much worse	☐
2	worse	☐
3	equal	☐
4	better	☐
5	much better	☐
Please justify your answer by indicating PROs and CONs		

Cadière Forceps		
1	much worse	☐
2	worse	☐
3	equal	☐
4	better	☐
5	much better	☐
Please justify your answer by indicating PROs and CONs		

Needle Driver		
1	much worse	☐
2	worse	☐
3	equal	☐
4	better	☐
5	much better	☐
Please justify your answer by indicating PROs and CONs		
